# Polyethylenimine-modified graphene quantum dots promote endothelial cell proliferation

**DOI:** 10.1093/rb/rbae013

**Published:** 2024-02-24

**Authors:** Qirong Xu, Chen Li, Xiangyan Meng, Xinghong Duo, Yakai Feng

**Affiliations:** School of Chemistry and Chemical Engineering, Qinghai University for Nationalities, Xining 810007, PR China; Key Laboratory of National Ethnic Affairs Commission of Resource Chemistry and Ecological Environment Protection on Qinghai-Tibet Plateau, Xining 810007, PR China; School of Chemistry and Chemical Engineering, Qinghai University for Nationalities, Xining 810007, PR China; Key Laboratory of National Ethnic Affairs Commission of Resource Chemistry and Ecological Environment Protection on Qinghai-Tibet Plateau, Xining 810007, PR China; Institute of Disaster and Emergency Medicine, Tianjin University, Tianjin 300072, PR China; School of Chemistry and Chemical Engineering, Qinghai University for Nationalities, Xining 810007, PR China; Key Laboratory of National Ethnic Affairs Commission of Resource Chemistry and Ecological Environment Protection on Qinghai-Tibet Plateau, Xining 810007, PR China; School of Chemical Engineering and Technology, Tianjin University, Tianjin 300350, PR China; Key Laboratory of Systems Bioengineering (Ministry of Education), Tianjin University, Tianjin 300072, PR China

**Keywords:** graphene quantum dots, polyethylenimine, ZNF580 gene, endothelial cell, proliferation

## Abstract

Endothelial cell proliferation plays an important role in angiogenesis and treatment of related diseases. The aim of this study was to evaluate the effect of polyethylenimine (PEI)-modified graphene quantum dots (GQDs) gene vectors on endothelial cell proliferation. The GQDs-cationic polymer gene vectors were synthesized by amidation reaction, and used to deliver pZNF580 gene to Human umbilical vein endothelial cells (HUVECs) for promoting their proliferation. The chemical modification of GQDs can adjust gene vectors’ surface properties and charge distribution, thereby enhancing their interaction with gene molecules, which could effectively compress the pZNF580 gene. The CCK-8 assay showed that the cell viability was higher than 80% at higher vector concentration (40 μg/mL), demonstrating that the GQDs-cationic polymer gene vectors and their gene complex nanoparticles (NPs) having low cytotoxicity. The results of the live/dead cell double staining assay were consistent with those of the CCK-8 assay, in which the cell viability of the A-GQDs/pZNF580 (94.38 ± 6.39%), C-GQDs-PEI- polylactic acid-co-polyacetic acid (PLGA)/pZNF580 (98.65 ± 6.60%) and N-GQDs-PEI-PLGA/pZNF580 (90.08 ± 1.60%) groups was significantly higher than that of the Lipofectamine 2000/pZNF580 (71.98 ± 3.53%) positive treatment group. The results of transfection and western blot experiments showed that the vector significantly enhanced the delivery of plasmid to HUVECs and increased the expression of pZNF580 in HUVECs. In addition, the gene NPs better promote endothelial cell migration and proliferation. The cell migration rate and proliferation ability of C-GQDs-PEI-PLGA/pZNF580 and N-GQDs-PEI-PLGA/pZNF580 treatment groups were higher than those of Lipofectamine 2000/pDNA treatment group. Modified GQDs possess the potential to serve as efficient gene carriers. They tightly bind gene molecules through charge and other non-covalent interactions, significantly improving the efficiency of gene delivery and ensuring the smooth release of genes within the cell. This innovative strategy provides a powerful means to promote endothelial cell proliferation.

## Graphical abstract

**Figure:**
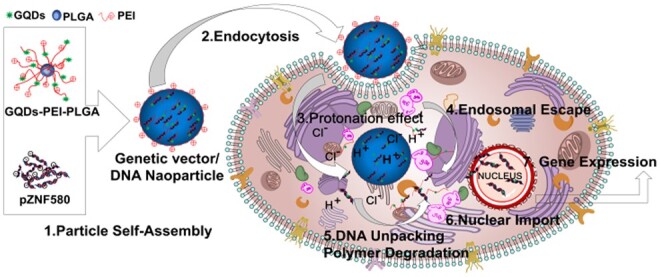

## Introduction

Cardiovascular disease is a major cause of high mortality, so tissue-engineered blood vessels (TEBVs) have great potential for coronary artery bypass grafting [1]. Due to the lack of functional endothelium [2], TEBVs constructed by conventional methods are prone to endothelial proliferation and thrombosis problems after transplantation [3]. An intact endothelial cell layer maintains vascular structure and ensures long-term patency of the TEBV [4]. Therefore, promoting endothelial cell proliferation and migration is a key strategy to solve the problems after TEBV transplantation [5].

Currently, methods commonly used for TEBV endothelialization include surface modification [6] and gene therapy [7]. Surface modification involves modifying the surface of vascular materials with substances such as heparin [8], polyethylene glycol [9], functional peptides [10] and biologically active substances [11], which are designed to promote the adhesion and growth of HUVECs [12]. Gene therapy, on the other hand, safely and effectively delivers target genes to target cells through gene vectors to promote cell proliferation and differentiation to form a complete endothelial cell layer [13], which has the advantages of accuracy, durability, controllability and low toxicity [14].

With the deepening of cardiovascular disease research, vascular endothelial growth factor (VEGF) [15], angiopoietin (Ang) [16], fibroblast growth factor (FGF) [17] and zinc-finger nuclear transcription factor gene (ZNF580) [18] have been found to promote the proliferation and migration of HUVECs. Among them, ZNF580 gene overexpression not only significantly enhanced HUVEC migration and proliferation [19], but also inhibited smooth muscle cell proliferation [20] and promoted endothelial progenitor cell differentiation to HUVECs [21], and enhanced angiogenesis through the eNOS/NO pathway [22].

Gene vectors carry target genes to form gene NPs, which are endocytosed into cells through the cell membrane, and then escape from endosomes/lysosomes, and finally the target genes enter into the cell nucleus and are successfully expressed, thus realizing the regulation of specific genes [23, 24]. In order to ensure that gene vectors can effectively deliver genes, they should have appropriate particle size, good stability, high transfection efficiency and low cytotoxicity [25], and the ideal particle size should be between 100 and 200 nm for the smooth entry of NPs into the cell and prolonged circulation time [26]. Polyethylenimine (PEI) of 25 kDa is recognized as the ‘gold standard’ for non-viral gene vectors [27]. Its multistage amine structure is positively charged and binds stably to DNA and RNA [28]. In the lysosomal low-pH environment, PEI can trigger the proton sponge effect and help nucleic acid molecules to escape [29]. High molecular weight PEIs are efficient but toxic, while low molecular weights are safe but less effective [30]. Previously, we prepared biodegradable amphiphilic block copolymer micelles using low molecular weight PEI (1800 Da) and PLGA [31]. This not only reduced the toxicity of PEI, but also improved the transfection efficiency [32]. PLGA has good biocompatibility and degradability and can be gradually converted to lactic acid and pyruvic acid *in vivo* and excreted through normal metabolic pathways [33]. Such micelles form a core-shell structure with PLGA as the core and PEI as the shell, which enhances carrier stability and prolongs in vivo circulation time [34].

Quantum dots have been widely studied in the field of tissue engineering [35] and can be used in bioimaging [36], biosensing [37], tissue repair [38] and functional delivery carriers [39]. Graphene quantum dots (GQDs) are novel carbon nanomaterials with graphene-like sp2 structure [40], with low toxicity and good biocompatibility [41], and no negative impact on cell viability and proliferation [42], and are non-toxic in vivo and ex vivo [43]. GQDs tightly bind to genes through non-covalent bonds, such as π–π stacking and electrostatic interactions [44], to enhance the stability and functionality and to protect genes from external influences [45]. It also forms a stable structure with cationic polymers [46], which can efficiently deliver genes to target cells and improve the transfection efficiency [47]. Liu et al [48] developed a GQDs-based gene delivery system, which realized safe *in vivo* delivery of atherosclerotic plaque gene drugs by cross-linking RNAs with GQDs and protecting miRNAs from degradation.

Quantum dots are naturally endowed with the ability to act as carriers due to their unique structural and physical properties [49]. By combining them with cationic polymers, efficient gene delivery and transport processes can be realized [50]. In this paper, a core-shell structured vector with PLGA as the core and GQDs-PEI as the shell was prepared by using the amidation reaction between PEI (10 KDa), PLGA and GQDs [51]. The binding ability of the gene vector to the pZNF580 gene and its cytotoxicity magnitude to HUVECs were investigated by cellular experiments, and the transfection, migration and proliferation efficiency of the NPs on HUVECs were also explored [52, 53].

## Experiment

### Main reagents and instruments

The Amino-graphene quantum dots (A-GQDs), Carboxylated graphene quantum dots (C-GQDs) and Nitrogen-doped graphene quantum dots (N-GQDs) used in the experiment were purchased from Jiangsu Xianfeng Nanomaterials Technology Co., LTD. Branched PEI (10 kDa), 1-ethyl-(3-dimethylaminopropyl) carbodiimide hydrochloride (EDC), N-hydroxysuccinimide (NHS) and carboxylated poly (lactic-co-glycolic acid) (PLGA-COOH) were purchased from Shanghai Dipak Chemical Technology Co., LTD.

The functional groups of the carrier materials were determined using a Fourier transform infrared (FTIR, iS5N, America) spectrometer. A transmission electron microscope (TEM, JEOL-2100F, Japan) was used to test the morphology of the vectors. The surface potential and particle size distribution of GQDs and gene vectors were measured by dynamic light scattering (DLS) instrument (Malvern, ZS90, UK). CKK-8 assay was performed using a microplate reader (Abbkine, BMU106-CN, China). The binding and assembly ability of the GQDs-PEI system with pZNF580 was detected and evaluated using gel electrophoresis apparatus (Bio-Rad, 5200, China). A Nikon inverted fluorescence microscope (Nikon-Eclipse, Ts2-FL, Japan) and an automatic chemiluminescence fluorescence image analyzer (Tanon-5200Multi, China) were used to record cell imaging, migration and proliferation.

### Construction of pIRES2-ZNF580-EGFP (pZNF580) plasmid

The pZNF580 plasmid was supported by technical services provided by GENEWIZ (Suzhou, China). The 5' (EcoRI) and 3' (BamHI) were added to the synthesized ZNF580 gene, and the gene was cloned into the vector pIRES2-EGFP (Kanamycin) via 5' EcoRI and 3' BamHI to construct the pIRES2-ZNF580 with green fluorescent protein (EGFP, Ex/Em = 488/507 nm) -EGFP (pZNF580) plasmid.

### Experimental methods

#### Preparation of C-GQDs-PEI

EDC and NHS were used to active the amidation reaction of C-GQDs and branched PEI. The reaction was carried out at room temperature (RT) under stirring for 24 h, followed by dialysis and freeze-drying to finally obtain a solid sample of C-GQDs-PEI.

#### Preparation of C-GQDs-PEI-PLGA

EDC and NHS were added to the C-GQDs solution (200 µg/mL) with a molar ratio of 1:2:2.2, and the solution was kept at RT for 3 h. PEI was added to the solution at a molar ratio of 1:1, and the reaction was continued for another 24 h. During this period, 400 µg/mL PLGA solution was activated by EDC/NHS with a molar ratio of 1:2:2.2 for 2 h. Finally, the activated PLGA solution was added to the reaction system and the reaction was continued for 24 h. After completion of the reaction, dialysis treatment was performed, and the dialysis time was more than 24 h. Finally, freeze-drying was performed to obtain the final solid sample of C-GQDs-PEI-PLGA.

#### Preparation of N-GQDs-PEI-PLGA

According to the preparation method of C-GQDs-PEI-PLGA, N-GQDs-PEI-PLGA was also prepared from nitrogen-doped GQDs.

### Chemical characterization of gene vectors

GQDs-cationic polymer gene vectors were characterized by FTIR, DLS, TEM and photoluminescence spectroscopy (PL).

#### FTIR spectroscopy analysis

Five to ten  milligrams of gene vectors were weighed and analyzed on a FTIR spectrometer to detect the functional groups with a scanning range of 400–4000 cm^−1^.

#### DLS 

The hydrodynamic diameter and zeta potential of the gene vectors and their complexes were measured by DLS.

#### TEM

The gene vectors were diluted to 1 mg/mL solution with absolute ethanol, and the solution was dispersed by an ultrasonic disperser. The dispersed solution was dropped onto the ultra-thin carbon film copper net and dried by oven, and the above steps were repeated for 3 times. After the copper net was completely dried, TEM was used for detection.

#### PL

A certain concentration of gene carrier aqueous solution was prepared with a slit width of 2 nm and a spectral range of 400–600 nm to measure fluorescence emission spectrum.

### Biological characterization of complexes NPs

The biological characteristics of the complexes NPs were analyzed by cell culture, Agarose gel electrophoresis, CCK-8 experiment, live/dead cell double staining experiment, cell transfection experiment, western blot, Scratch test and EdU incorporation test.

#### Preparation of pZNF580 complexes NPs

The pZNF580 plasmid was diluted to 200 μg/mL with PBS (pH = 7.4) buffer. The gene vectors solution (0.25 mg/mL) was prepared, and then the pZNF580 plasmid was added to the gene vectors solution at different w/w weight ratios (w_NPs_/w_pZNF580_ = 0.25, 0.5, 1, 2, 3, 4) and mixed. The pZNF580 complexes NPs were prepared by standing for 30 min at room temperature.

#### Cell culture

HUVECs were cultured using endothelial cell medium (ScienCell, 1001, USA) in a cell culture incubator (37°C, 5% CO_2_). Cells were collected by centrifugation after digestion with trypsin when the cell growth fusion reached 80–90%, and then the medium was added and mixed, a certain amount of cells were counted, and plates were spread for experiments.

#### Agarose gel electrophoresis experiment

NPs solutions with different w/w weight ratios (w_NPs_/w_pZNF580_ = 0.25, 0.5, 1, 2, 3, 4) were prepared and then mixed with 6× sampling buffer (2 μL) in agarose gel (0.80 wt %) containing 0.50 μg/mL nucleic acid dye (Goldview). Electrophoresis was performed for 35 min at 100 V in 1× TAE buffer. Analysis was performed in a gel imager (Tanon 5200) to determine the position of the vectors when fully loaded with the gene.

#### CCK-8 experiment

HUVECs were seeded into 96-well plates (1 × 10^4^ cells/well) and placed in the incubator for 24 h to 80–90% confluence. Then, different concentrations of NPs were added to the cell culture plate, which were 5, 10, 20, 40 and 60 µg/mL, respectively. To ensure the accuracy and reliability of the results, three replicate wells were set up for each concentration, i.e., three separate sample wells for each concentration condition. After 48 h of culture, 10 µL of CCK-8 reagent (Abbkine, BMU106-CN, China) was added to each well and incubated in the incubator for 1 h, and the absorbance value was measured at 450 nm by a microplate reader. Relative cell viability (%) was calculated using the following formula:

$$\text{Relative cell viability}(\%)=\frac{OD-{\text{OD}}_{0}}{OD_{1}-OD_{0}}\times 100\%$$

where OD is the absorbance of the cells treated in the experimental group, OD_1_ is the absorbance of the cells without any treatment, and OD_0_ is the absorbance value of the zerosetting well with only medium and CCK-8.

#### Live/dead cell double staining experiment

The proliferation of HUVECs was analyzed using a live (liveDye; Ex/Em = 488/530 nm)/dead (DeadDye; Ex/Em = 535/617 nm) cell double staining kit (Abbkine, KTA1001, China). HUVECs with good growth status were taken and seeded in 48-well plates at a density of 1 × 10^4^cells/well and incubated until 50–70% fusion. Different complexes were utilized for the administration of treatments containing 1 µg of pZNF580 plasmid per well. The incubation was continued using medium with 2% FBS for 48 h. The medium was discarded, washed three times with PBS and replaced with fresh medium and live/dead cell double staining reagent working solution to continue the incubation for 15 min. Photographs were taken and recorded using the inverted fluorescence microscope. The green mean fluorescence intensity was statistically analyzed using Image J software.

#### In vitro transfection experiments

HUVECs were seeded in 24-well plates at a density of 4 × 10^4^ cells/well and incubated overnight in an incubator until 80–90% confluence. Before transfection, cells were starved in serum-free DMEM medium for 8 h. After that, different NPs gene complexes were added to 24-well plates containing 1 μg of plasmid per well. The incubation was continued at 37°C in 5% CO_2_ for 4 h to 6 h, then replaced with complete medium and placed in an incubator to continue the culture. At 36 h, the transfection of cells was observed by inverted fluorescence microscope and photographed for recording.

#### Western blot experiments

The transfection effect of NPs and the protein expression level of ZNF580 were analyzed by western blot (Beyotime, P0012S, China).

Protein extraction: HUVECs transfected with NPs were cultured, and the cells were lysed and the supernatant was collected.

Protein denaturation: After adding gel loading buffer, the protein samples were denatured by high temperature treatment.

Protein separation by electrophoresis: The protein samples were placed in SDS-PAGE gel and separated by electrophoresis.

Protein transfer to PVDF membrane: The gel was cut and immersed in the transfer solution, and then the PVDF membrane was trimmed to the appropriate size and activated. The PVDF membrane was stacked with gel and filter paper for membrane transfer.

For protein hybridization, the transformed PVDF membrane was immersed in protein blocking solution, and then diluted primary and secondary antibodies were added and incubated under appropriate conditions.

Development: After washing the films, the ECL developer solution was used for development, photography was performed by gel imager, and statistical analysis of protein expression was performed using Image J software.

#### Cell scratch test

HUVECs were seeded into six-well plates (4 × 10^5^ cells/well) and incubated in the incubator until the cells were covered with the bottom of the wells. Then, HUVECs cells were transfected with different NPs gene complexes in a six-well plate, and 24 h after transfection, a cell wound scratch was made in each well with a gun tip. The scratched cell debris in the wells was washed with D-hanks buffer. At different time points (0 h, 12 h and 24 h), the migration process of cells in the wound scratch was observed by the microscope. Then Image J software was used to count the migration area of the cells in the photographs. The specific calculation formula is as follows:

$$\text{Migration area}(\%)=\frac{S\;-\;S_{0}}{S}\;\times \;100\%$$

where S is the area of cell scratch at 0 h and S_0_ is the area of unhealed at 24 h.

#### EdU admixture assay

The EdU cell proliferation image kit (Abbkine, KTA 2031, China) was followed. HUVECs transfected with ZNF580 were incubated in EdU (10 μM) medium for 24 h. The cells were then fixed with 4% paraformaldehyde for 15 min. The fixative was removed and permeabilized with 0.5% Triton-X-100 for 15 min. The Click-iT reaction containing AbFluor 545 (Ex/Em = 546/565 nm) fluorescent dye was incubated for 30 min at room temperature. The mixture was incubated away from light at room temperature for 30 min. The reaction mixture was removed and subjected to nuclear staining (1×Hoechst 33342). Photographic documentation was performed using an inverted fluorescence microscope. The total number of orange and blue fluorescence was statistically analyzed using Image J software.

#### Statistical analysis

All statistical analyses were performed using Origin 2022 and StatsDirect software. ZNF580 protein, average fluorescence intensity of cells, relative cell migration area and *in vitro* tube formation length and number of nodes were quantified using Image J software. The experiments were repeated three times, and the experimental data were expressed as Mean ± SEM. Differences between the two groups were analyzed using one-way analysis of variance. *P < 0.05, ^#^P < 0.05 were considered statistically significant, and ‘ns’ indicated no statistical significance.

## Results and discussion

### Chemical characterization of gene vectors

**Figure 1.**
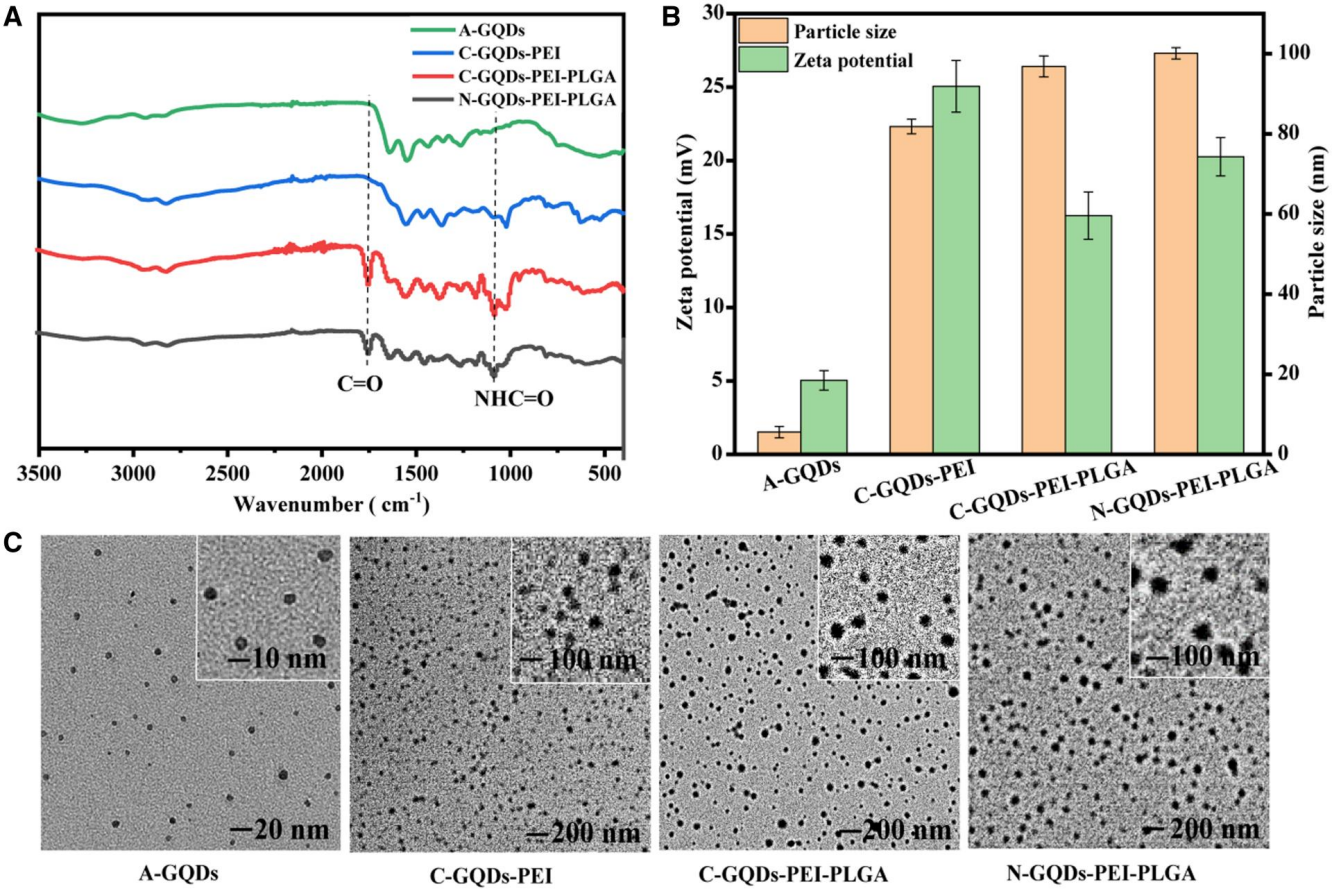
FTIR (**A**), average particle size and zeta potential distributions (**B**) and TEM images (**C**) of A-GQDs, C-GQDs-PEI, C-GQDs-PEI-PLGA and N-GQDs-PEI-PLGA.

The characteristic functional groups of the gene vectors were detected by FTIR spectroscopy (Figure 1A). The results showed -OH stretching vibration at 3358 cm^−1^, stretching and bending vibration attributed to N-H bond at 3150 cm^−1^, and C-N bending vibration at 1283 cm^−1^. FTIR spectrum analysis showed that there were functional groups such as -C=O, -OH, -NH and -NH_2_ on the surface of A-GQDs. The characteristic peak of -CONH amide bond around 1700 cm^−1^ and 1100 cm^−1^ after PEI and PLGA grafting indicated successful grafting.

Figure 1B shows the average particle size and average potential of A-GQDs and vectors of C-GQDs-PEI, C-GQDs-PEI-PLGA and N-GQDs-PEI-PLGA. The average particle size of A-GQDs was 5.57 ± 1.42 nm, while the average particle size of C-GQDs-PEI, C-GQDs-PEI-PLGA and N-GQDs-PEI-PLGA vectors was 81.83 ± 1.81 nm, 96.84 ± 2.61 nm and 100.10 ± 1.43 nm, respectively. The A-GQDs had the average zeta potential of 5.04 ± 0.66 mV, while C-GQDs-PEI showed a relative high zeta potential (25.06 ± 1.76 mV). PLGA led to a slight decrease in zeta potential, namely C-GQDs-PEI-PLGA (16.25 ± 1.61 mV) and N-GQDs-PEI-PLGA (20.26 ± 1.31 mV). The vectors with positive charge is conducive to endocytosis and transfection.

Figure 1C shows the TEM images of A-GQDs, C-GQDs-PEI, C-GQDs-PEI-PLGA and N-GQDs-PEI-PLGA. It can be seen that each sample is distributed very uniformly, without large nanoparticles or aggregation, and has uniform shape and size.

**Figure 2.**
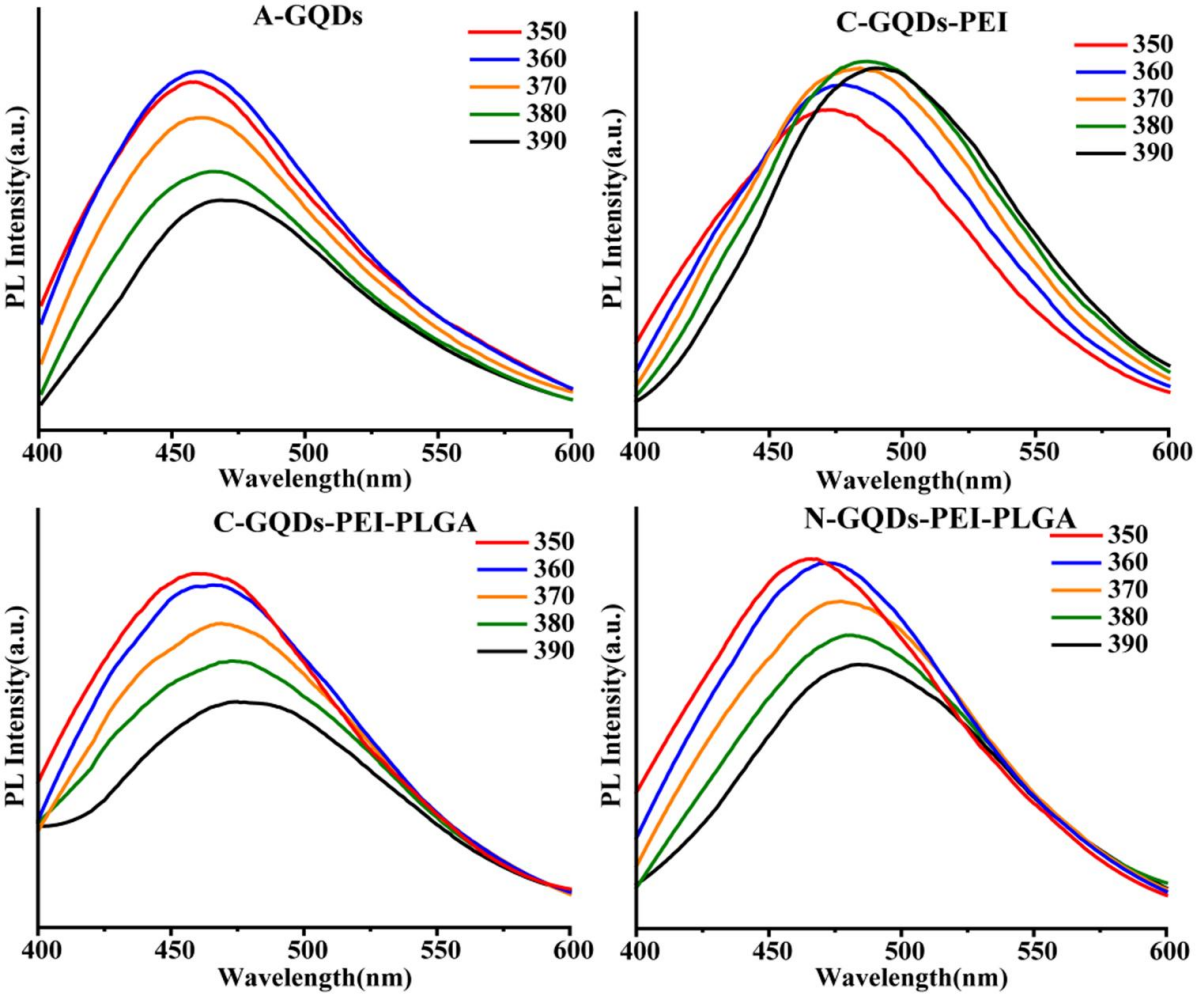
Steady-state fluorescence spectra of A-GQDs, C-GQDs-PEI, C-GQDs-PEI-PLGA and N-GQDs-PEI-PLGA.

The steady-state fluorescence spectrum of A-GQDs, C-GQDs-PEI, C-GQDs-PEI-PLGA and N-GQDs-PEI-PLGA were excited by 350-390 nm excitation light (Figure 2). A-GQDs have an excitation wavelength dependence. The excitation wavelength at 360 nm showed the maximum fluorescence intensity of A-GQDs was maximum at the emission wavelength of 460 nm. When the excitation wavelength was 380 nm, the fluorescence intensity of C-GQDs-PEI was maximum at the emission wavelength of 480 nm. When the excitation wavelength was 350 nm, the fluorescence intensity of C-GQDs-PEI was maximum at 460 nm, but the fluorescence intensity of N-GQDs-PEI emission wavelength was the maximum at 465 nm. The fluorescence emission peak FWHM was small and the peak shape was symmetrical, indicating that the C-GQDs in the C-GQDs-PEI, C-GQDs-PEI-PLGA and N-GQDs-PEI-PLGA had narrow size distribution.

### Biological characterization of complexes NPs

#### Encapsulating capacity of gene vectors to pZNF580 and biocompatibility of complexes NPs

**Figure 3.**
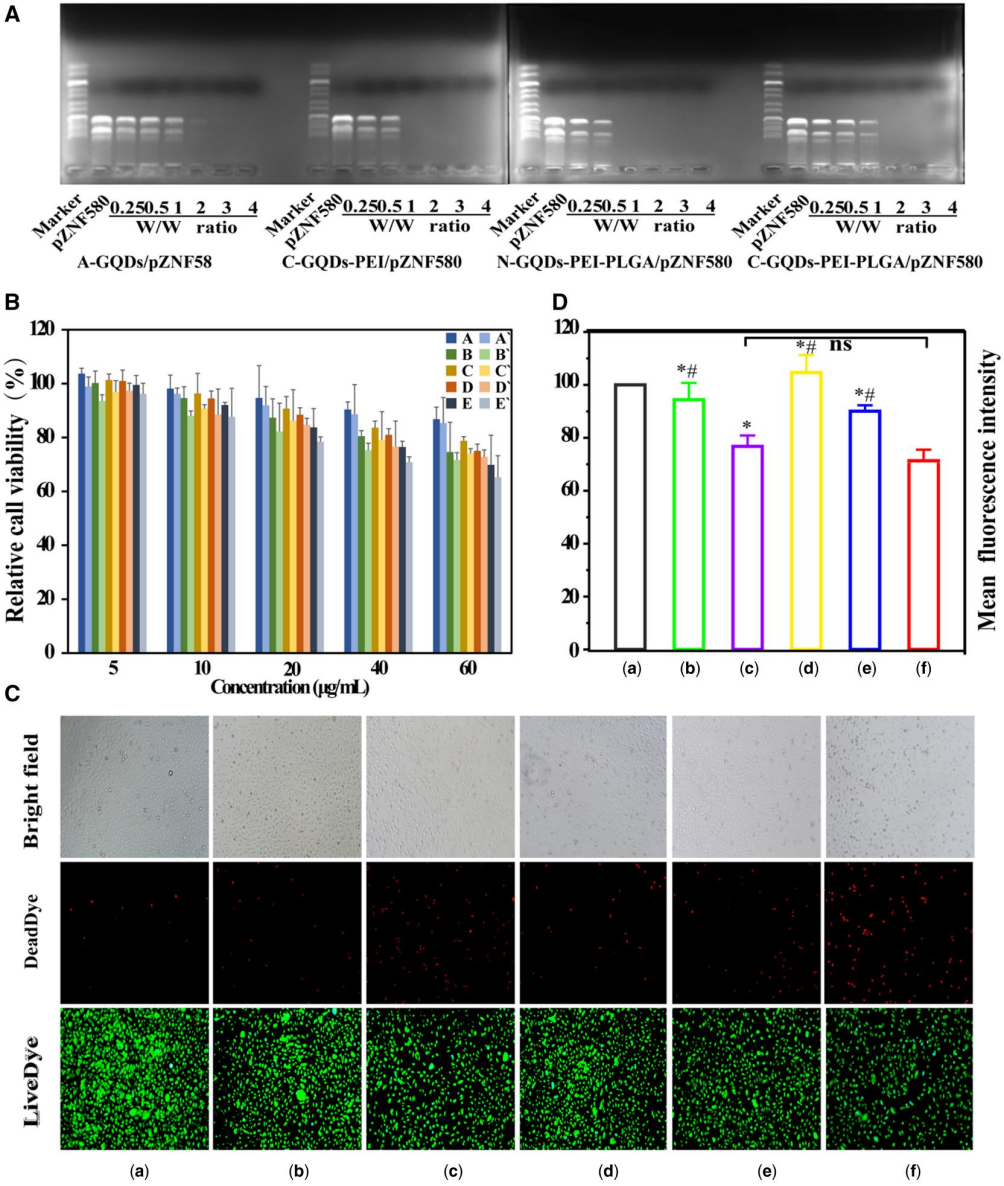
Agarose gel electrophoregrams of different NPs at different weight ratios (**A**); the relative cell viability of HUVECs with different gene vectors and NPs for 48 h by CCK-8 assay (**B**); (a) A-GQDs/pZNF580 treatment group, (a′) A-GQDs treatment group, (b) C-GQDs-PEI/pZNF580 treatment group, (b′) C-GQDs-PEI treatment group, (c) C-GQDs-PEI-PLGA/pZNF580 treatment group, (c′) C-GQDs-PEI-PLGA treatment group, (d) N-GQDs-PEI-PLGA/pZNF580 treatment group, (d′) N-GQDs-PEI-PLGA-treated group, (e) Lipofectamine 200 0/pZNF580 treatment group, (e′) Lipofectamine 2000 treatment group. The relative cell viability of HUVECs with different gene vectors and NPs for 48 h by live/dead cell double staining assay (**C**) and relative cell viability of HUVECs with different composite gene vectors and NPs for 48 h by Image J (**D**). (a) pZNF580, (b) A-GQDs-PEG-CAG/pZN F580, (c) C-GQDs-PEI-PEG-CAG/pZNF580, (d) C-GQDs-PEI-PLGA-PEG-CAG/p ZNF580, (e) N-GQDs-PEI-PLGA-PEG-CAG/pZNF580, (f) Lipofectamine 2000/pZ NF580. (Mean ± SEM, n = 3, *P < 0.05 VS a, ^#^P < 0.05 VS F).

The ability of the gene vectors to encapsulate pZNF580 was investigated by agarose gel electrophoresis. As shown in Figure 3A, the pZNF580 with a negative charge can migrate from the negative to the positive electrode in the electrophoretic condition. Some or all pZNF580 was blocked when the pZNF580 was compressed by different vectors. A-GQDs were loaded with pZNF580 at a weight ratio of 3. C-GQDs-PEI and N-GQDs-PEI-PLGA could be fully loaded with pZNF580 at a weight ratio of 1. However, the C-GQDs-PEI-PLGA can fully load pZNF580 when the weight ratio was 2. Because the introduction of PLGA reduces the zeta potential of C-GQDs. For more efficient compression of pZNF580, a higher zeta potential is required. However, it is well known that gene vectors usually exhibit relatively high cytotoxicity when large amounts of positive charges are present on their surface. Considering these two aspects, a weight ratio of 3 was chosen for study in later experiments.

The cytotoxicity of gene vectors and NPs *in vitro* were initially detected by CCK-8 assay. Lipofectamine 2000 and Lipofectamine 2000/pZNF580 were used as positive treatment group. As shown in Figure 3B, the cytotoxicity of different gene vectors and NPs increased with the increase of vector concentration. At the same concentration, compared with the positive treatment group of Lipofectamine 2000 and Lipofectamine 2000/pZNF580, the cell viability of different gene vectors and NPs groups was relatively higher. At the same concentration, compared with A-GQDs, the cell activity of the PEI-containing complex group was lower, which was due to the large amount of positive charge in PEI. However, the introduction of PLGA reduced the toxicity of C-GQDs-PEI and N-GQDs-PEI gene carriers. Even at a higher concentration of vector (40 μg/mL), the cell viability of gene vectors and NPs was still over 80%. These results suggest that these vectors showed low cytotoxicity.

To further verify the above results, a live/dead cell double-staining experiment was performed, which was able to visualize the survival status of the cells. After treating HUVECs with different NPs for 48 h (Figure 3C), all NPs/pZNF580 HUVECs showed high cellular activity compared to the negative treatment group (pZNF580), whereas the number of viable cells in the Lipofectamine 2000/pZNF580 positive treatment group was significantly lower than that in the test group (pZNF580), indicating the high cytotoxicity of Lipofectamine 2000. Statistical analysis of the mean cell green fluorescence intensity values revealed (Figure 3D) that, compared with the Lipofectamine 2000/pZNF580 (71.98 ± 3.53%) positive treatment group, A-GQDs/pZNF580 (94.38 ± 6.39%), C-GQDs-PEI-PLGA/pZNF580 (98.65 ± 6.60%) and N-GQDs-PEI-PLGA/pZNF580 (90.08 ± 1.60%)-treated HUVECs had a higher average fluorescence intensity, which was attributed to the greater toxicity of Lipofectamine 2000 to the cells, resulting in a massive cell death. In addition, C-GQDs-PEI-PLGA/pZNF580 exhibited a higher cell viability. These results corresponded to the results of CCK-8 experiments.

#### Cell vitro transfection and ZNF580 expression

**Figure 4.**
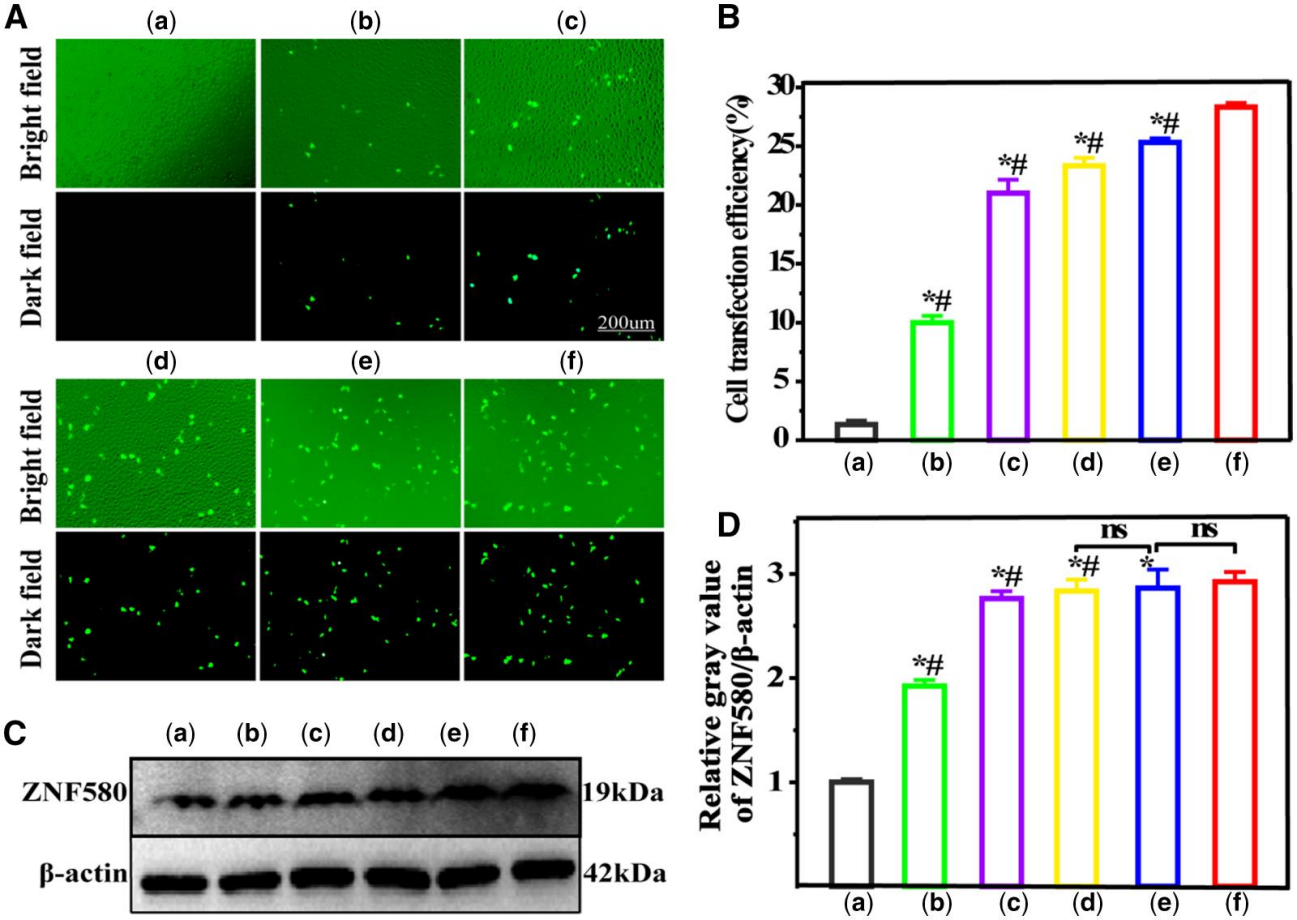
Fluorescence plots (**A**) and transfection efficiency plots (**B**) after transfection with different NPs at 24 h. Western blot of ZNF580 protein expression in HUVECs cells transfected with NPs (**C**) and protein expression efficiency plots by Image J software (**D**) (w_NPs_/w_pZNF580 _= 3). (a) pZNF580 treatment group, (b) A-GQDs/pZNF580 treatment group, (c) C-GQDs-PEI/pZNF580 treatment group, (D) C-GQDs-PEI-PLGA/pZNF580 treatment group, (e) N-GQDs-PEI-PLGA/pZNF580 treatment group, (f) Lipofectamine 2000/pZNF580 treatment group. (Mean ± SEM, n = 3, *P < 0.05 VS a, ^#^P < 0.05 VS F).

To evaluate the transfection efficiency of the NPs, pZNF580 was used as the target gene to transfect HUVECs. HUVECs transfected with Lipofectamine 2000/pZNF580 were used as a positive treatment control, and pZNF580 without vector was used as a negative control. As shown in Figure 4A, naked pZNF580 was difficult to transfect HUVECs. In contrast, the gene vectors provided excellent transfection ability, indicating that they could facilitate the successful transfection of pZNF580 gene into cells and achieve expression. In addition, the transfection efficiency of N-GQD-PEI-PLGA/pZNF580 NPs was slightly lower than that of the Lipofectamine 2000/pZNF580 positive treatment control. The transfection efficiency of A-GQDs/pDNA without PEI was relatively lower than that of the other complexes, among which the transfection efficiency of C-GQDs-PEI/pDNA NPs was lower than that of C-GQDs-PEI-PLGA/pDNA NPs. These results indicated that GQDs modified by PEI could enhance its transfection efficiency to cells, and the introduction of PLGA increased the transfection efficiency.

Further, western blot experiments were used to detect the protein expression of the target gene ZNF580. As shown in Figure 4D, the protein expression of ZNF580 in the NPs transfection groups was significantly higher than that in the negative control group. Compared with the Lipofectamine 2000/pZNF580 positive treatment control, the protein expression of ZNF580 in the cells transfected with N-GQDs-PEI-PLGA/pZNF580 NPs was slightly higher. The protein expression of ZNF580 in cells transfected with A-GQDs/pDNA without PEI was relatively lower than other complexes. The protein expression of ZNF580 in C-GQDs-PEI/pDNA and C-GQDs-PEI-PLGA/pDNA NPs-transfected cells was close to that of the Lipofectamine 2000/pZNF580 positive treatment control. These results indicated that these NPs could significantly enhance plasmid delivery into HUVECs and enhance the expression of pZNF580 in HUVECs.

#### Cell migration and proliferation in vitro

**Figure 5.**
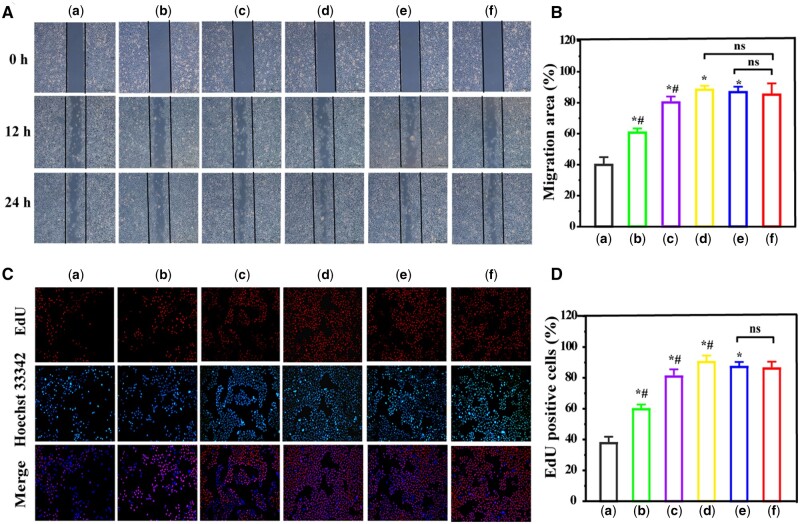
The migration process of HUVECs cells at different time points (**A**) and the relative percentage of cell migration area at 24 h calculated by Image J (**B**). Proliferation of HUVECs by NPs at 24 h determined by EdU doping exp eriment (**C**) and the total number of orange and blue fluorescence was statistically analyzed by Image J (**D**). (a) pZNF580 treatment group, (b) A-GQDs/pZNF580 treatment group, (c) C-GQDs-PEI/pZNF580 treatment group, (d) C-GQDs-PEI-PLGA/pZNF580 treatment group, (e) N-GQDs-PEI-PLGA/pZN F580 treatment group, (f) Lipofectamine 2000/pZNF580 treatment group. (Mean ± SEM, n = 3, *P < 0.05 VS a, ^#^P < 0.05 VS F).

The migration ability of the cells was measured by scratch assay, and the results are shown in Figure 5A. Cells treated with Lipofectamine 2000/pDNA served as a positive control and cells treated with pZNF580 served as a negative control. The NPs with PEI-modified GQDs showed a better migration rate compared with the negative control. The cell migration rates of the C-GQDs-PEI-PLGA/pZNF580 (88.35 ± 0.72%) and N-GQDs-PEI-PLGA/pZNF580 (86.83 ± 1.55%) treatment groups were similar and did not differ significantly from the positive control Lipofectamine 2000/pDNA (85.18 ± 4.19%). There was no significant difference. It was slightly higher in the C-GQDs-PEI-PLGA/pZNF580 treatment group compared to the C-GQDs-PEI/PLGA/pZNF580 treatment group, suggesting that the introduction of PLGA improved its cytocompatibility. These results suggest that GQDs-cationic polymer gene carrier/pZNF580 NPs can enhance the migration of HUVECs.

The effect of NPs on the proliferation of HUVECs was further analyzed using the EdU method. The results showed that proliferation of HUVECs occurred in all groups, and all NPs significantly promoted the proliferation of HUVECs (Figure 5C). The cell proliferation rate was slightly lower in the C-GQDs-PEI/pZNF580 group (80.79 ± 2.57%) compared to the Lipofectamine 2000/pZNF580 positive control group (86.05 ± 2.31%). However, both the C-GQDs-PEI-PLGA/pZNF580 group (90.29 ± 2.75%) and the N-GQDs-PEI-PLGA/pZNF580 group (87.05 ± 2.48%) showed significantly higher cell proliferation capacity than that of the Lipofectamine 2000/pZNF580 positive control group. Specifically, the C-GQDs-PEI-PLGA/pZNF580 group showed the highest cell proliferation rate of 90.29 ± 2.75%, whereas the N-GQDs-PEI-PLGA/pZNF580 group also exhibited a higher cell proliferation capacity of 87.05 ± 2.48%. These results indicated that C-GQDs-PEI-PLGA and N-GQDs-PEI-PLGA nanocarriers possessed higher cell proliferation-promoting effects than Lipofectamine 2000 in delivering the pZNF580 gene (Figure 5D). Since the transfection efficiency of C-GQDs-PEI/pZNF580 was lower compared to Lipofectamine 2000/pZNF580, it resulted in a corresponding decrease in the expression level of ZNF580. In contrast, the C-GQDs-PEI-PLGA/pZNF580 and N-GQDs-PEI-PLGA/pZNF580 groups showed a similar expression level of ZNF580 and a higher biocompatibility with Lipofectamine 2000/pZNF580. In addition, the introduction of PLGA led to a decrease in the C-GQDs-PEI charge number, which enhanced cytocompatibility and resulted in C-GQDs-PEI-PLGA/pZNF580 and N-GQDs-PEI-PLGA/pZNF580 maintaining a high level of transfection and having a better level of cell proliferation.

According to the experimental results, the GQDs-cationic polymer vectors have good compression and encapsulation ability, biocompatibility, gene transfection and expression effect, and can significantly improve the migration and proliferation of HUVECs. Therefore, this gene carrier system is expected to be an effective means for the treatment of angiogenesis and related diseases.

The GQDs-cationic polymer gene vectors promote HUVECs proliferation, which may involve multiple aspects. Firstly, GQDs have a large specific surface area and excellent biocompatibility, which can provide a well cell adhesion microenvironment and growth conditions to promote the attachment and proliferation of HUVECs. Moreover, the high positive charge of GQDs may facilitate cell signaling and the regulation of bioactive molecules, thereby further promoting the proliferation and endothelialization process. Second, the high activity of PEI could provide an affinity within the cell that drives the binding of gene molecules to ribonucleases in the nucleus and the formation of stable complexes that protect the genes from degradation. Through its positive charge characteristics, ionization ability, transfection efficiency and intracellular release, the transfection and expression of foreign genes can be realized. In addition, the introduction of PLGA also improved the regulability and stability of gene vectors. The degradation of PLGA begins in response to the acidic environment within the cell or the action of enzymes, leading to the gradual breakdown of the gene vectors. This degradation ability allows the vectors to release the loaded genes, allowing them to enter the cytoplasm or even the nucleus.

GQDs-cationic polymer gene vectors showed positive result in gene delivery for HUVECs proliferation; however, there are still some issues that need further study to address. First, the toxicity and long-term stability of GQDs in HUVECs are relatively understudied and need further evaluation. In addition, the specific mechanism and function of GQDs-cationic polymer gene vectors are not fully understood, and further basic research is needed to reveal the mechanism of action. The current research mainly focused on *in vitro* cell experiments, and there is a lack of *in vivo* experimental evidence. Animal experiments are needed to verify its feasibility and effectiveness in TEBVs.

Based on the current research status, the following directions can be taken in the future study:

1. to further investigate the toxicity and biological safety of GQDs, including the effects on HUVECs function, cell apoptosis and inflammatory response.
2. to further study the mechanism of the GQDs modified gene vectors, including an in-depth analysis of the regulatory mechanism of GQDs on cell signaling pathways and gene expression.
3. to expand the scope of research and conduct more in vivo experimental evidence to evaluate the feasibility and effectiveness of the GQDs modified gene vectors.

## Conclusion

In this study, we successfully delivered the pZNF580 into HUVECs using the GQDs-cationic polymer vectors, which effectively promoted the proliferation and migration of HUVECs. To summarize the experimental results, C-GQDs-PEI, C-GQDs-PEI-PLGA and N-GQDs-PEI-PLGA have good gene-carrying capacity, of which N-GQDs-PEI-PLGA has the highest migration rate although its cytotoxicity is slightly higher than that of the other materials, and its transfection efficiency and protein expression are close to those of Lipofectamine 2000, which has the most development potential. The experimental results show that GQDs have excelent physicochemical properties and biocompatibility, providing a good adhesion environment and growth conditions for HUVECs. The gene vectors can help reduce the risk of thrombosis and improve long-term patency of TEBVs and have great potential for development in the field of TEBVs. In order to further optimize the vectors and improve therapeutic efficacy, we will conduct more in-depth studies in the future. Specifically, we will begin to investigate how to make the vectors more targetable and conduct a comprehensive safety evaluation of them. The gene vector provides a safe and effective solution for endothelialization of artificial blood vessels, an innovation that is expected to advance the field.
